# What Your Death Certificate Says About You May Be Wrong: A Narrative Review on CDC’s Efforts to Quantify Prescription Opioid Overdose Deaths

**DOI:** 10.7759/cureus.18012

**Published:** 2021-09-16

**Authors:** John F Peppin, John J Coleman, Antonella Paladini, Giustino Varrassi

**Affiliations:** 1 Osteopathic Medicine, Marian University, Indianapolis, USA; 2 Research and Development, DrugWatch International, Inc., Clifton, USA; 3 Anesthesiology and Pain Medicine, University of L'Aquila, L'Aquila, ITA; 4 Research, Paolo Procacci Foundation, Roma, ITA

**Keywords:** death certificate, centers for disease control and prevention (cdc), prescription opioid overdose deaths, illicitly manufactured fentanyl (imf), methadone, international classification of diseases (icd), benzodiazepines

## Abstract

Mortality data in most countries are reported using the International Classification of Diseases (ICD), managed by the WHO. In this paper, we show how the ICD is ill-suited for classifying drug-involved deaths, many of which involve polysubstance abuse and/or illicitly manufactured fentanyl (IMF).

Opioids identified in death certificates are categorized according to six ICD T-codes: opium (T40.0), heroin (T40.1), methadone (T40.3), other synthetic narcotics (T40.4), and other and unspecified narcotics (T40.6). Except for opium, heroin, and methadone, all other opioids except those that are unspecified are aggregated in two T-codes (T40.2 and T40.4), depending upon whether they are natural/semisynthetic or synthetic opioids other than methadone. The result is a system that obscures the actual cause of most drug overdose deaths and, instead, just tallies the number of times each drug is mentioned in an overdose situation.

We examined the CDC’s methodology for coding other controlled substances according to the ICD and found that, besides fentanyl, the ICD does not distinguish between other licit and illicitly manufactured controlled substances. Moreover, we discovered that the CDC codes all methadone-related deaths as resulting from the prescribed form of the drug. These and other anomalies in the CDC’s mortality reporting are discussed in this report.

We conclude that the CDC was at fault for failing to correct the miscoding of IMF. Finally, we briefly discuss some of the public policy consequences of this error, the misguided focus by public health and safety officials on pharmaceutical opioids, their prescribers and users, and the pressing necessity for the CDC to reassess how it measures and reports drug-involved mortality.

## Introduction and background

Vital statistics provide an important measure of a nation’s health and welfare. In this report, we focus on mortality statistics compiled and reported by the Centers for Disease Control and Prevention (CDC). In 2019, the CDC reported a total of 2,854,838 deaths in the United States (US) [1]. Of this number, 173,040 deaths (6%) were "unintentional injury deaths," a category that includes falls, motor vehicle traffic deaths, and unintentional poisoning deaths (i.e., drug overdose deaths) [2]. The number of unintentional poisoning deaths reported by the CDC for 2019 was 71,130 [3]. While only 2.5% of the total deaths in 2019, unintentional poisoning deaths account for more than a third (38%) of all unintentional injury deaths. This percentage is likely to increase as CDC’s preliminary estimates for 2020 show 93,000 drug overdose deaths - an increase of 30.7% over the previous year [3].

The process of recording deaths begins with the death certificate, a document that has critical administrative and epidemiologic applications. It is used to settle estates, resolve insurance claims, terminate pensions and public benefits, identify causes of morbidity and mortality, establish public health policies, and inform the allocation and expenditure of public funds for such purposes [4]. In the US, registering individual births and deaths is a state responsibility, while the responsibility for compiling and publishing national vital statistics rests with the federal government, specifically the CDC [5].

William Farr, a 19th-century British registrar general, in his annual report of deaths in 1840, warned certifiers to be specific in recording cause of death descriptions and to avoid the use of vague statements like "sudden death," "natural death," "visitation of God," and "old age." These, he said, obscured the proximate cause - what he referred to as the "internal morbid process." [6]. Voiced more than 180 years ago, Farr’s words remain true today and apply to death certifiers in the US and more than a hundred nations of the world that use some form of the death certification process to register and report mortality.

Although not a focus of this paper, the recent COVID-19 pandemic and the unusual fluctuations in global mortality ascribed to the coronavirus have uncovered some of the limitations in the current systems used by nations reporting COVID-19 deaths. According to figures compiled by Johns Hopkins University of Medicine’s Coronavirus Resource Center, between the beginning of the pandemic and December 15, 2020, global COVID-19 deaths fluctuated from a low of 0.3 per 100,000 population in China to a high of 156.5 per 100,000 population in Belgium. The US reported 91.6 deaths per 100,000 during this period [7]. A statistical range of such magnitude suggests inconsistencies in how individual nations define and report COVID-19 mortality.

**Figure 1:**
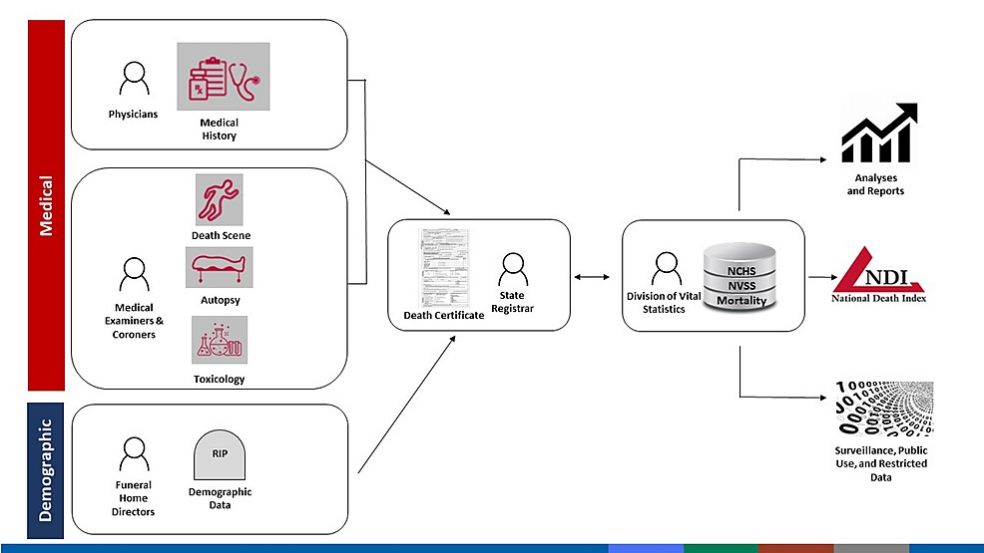
Vital statistics Source: CDC [10]

In the US, timelines and procedures for reporting and investigating accidental or suspicious deaths are set by state law and vary among states and even within states [8]. In the case of deaths that are neither accidental nor suspicious, the process of certifying the cause of death begins with the attending physician who is likely to have access to the decedent’s health record, including details of preexisting conditions that may have caused or contributed to death [9]. Upon completion and receipt by state registrars of vital statistics, portions of the death certificate, including the cause of death entries, are forwarded electronically to the CDC National Center for Health Statistics (NCHS) for inclusion in the National Death Index (Figure 1) [10].

One of the weakest links in this chain is at the beginning when the immediate and contributory causes of death are recorded by the certifying official. An error made or an incomplete cause of death at this juncture will pass from state to federal officials and result in a loss of data or worse, the corruption of the mortality database itself.

## Review

The US standard death certificate

As noted, the federal government has legal responsibility for annually publishing the nation’s vital statistics, including the manner and causes of deaths [11]. The US standard death certificate is the source document for compiling these statistics [11]. Since 1960, collecting this information has been the responsibility of the NCHS, a subdivision of the CDC [11,12]. The National Vital Statistics System (NVSS), a program within the NCHS, is responsible for receiving, compiling, and publishing this information [12].

Death certificate data, including cause(s) of death, are entered into the NVSS database by technicians using software that converts the literal language of the certifiers into category codes according to the WHO's International Classification of Diseases (ICD) [13]. Each year, the NVSS handles about 2.9 million death reports. They pass through an automated system that does not control for the accuracy or completeness of the data. Thus, the CDC begins its task of compiling national mortality data with raw source data that, according to the CDC’s estimate, arrives at the agency with an acknowledged error rate between 20-30% [14].

CDC guidelines for completing the standard death certificate require certifiers to list a single immediate cause of death in Section 32, Part I, along with a brief description of the sequence and timing of contributing causes leading up to the immediate cause of death. In Section 32, Part II, of the death certificate a certifier may include up to 20 contributory causes of death [13].

Over 20 years ago, an expert panel of state registrars and CDC officials proposed changes to the standard death certificate that eventually resulted in the creation of the electronic death registration system (EDRS) [15]. Being able to file a death certificate electronically greatly improved the efficiency of the process, but did little to improve accuracy. Hanzlick, a noted forensic pathologist, described this process as follows: "If a cause of death is stated improperly or not clearly, the person who classifies and codes the cause of death (a nosologist) uses a somewhat arbitrarily established system of rules to identify a cause of death for coding and official classification." [16]. 

The "arbitrarily established system of rules" Hanzlick mentioned is the ICD [16]. "Our national mortality statistics," Hanzlick cautioned, "are derived from codes, which, in turn, have been derived from causes of death as written on death certificates by certifiers. The value of an accurately and clearly stated cause of death to ensure proper coding and classification of deaths cannot be overstated." [16]. Although addressing present-day issues involving the EDRS and the ICD, Hanzlick’s concerns echoed those of 19th-century Registrar General Farr mentioned earlier.

**Figure 2:**
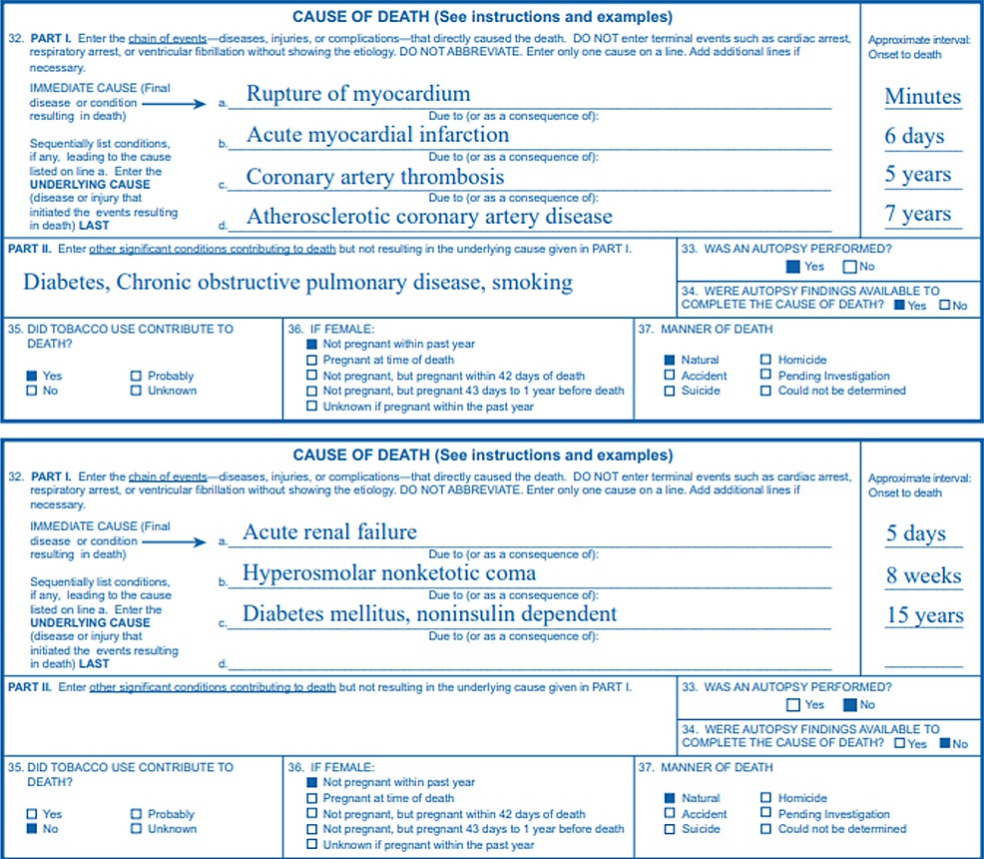
Example of a death certificate Source: CDC [17]; open-source

In 2004, the CDC published detailed instructions and examples for completing its revised death certificate [17]. Examples of the completed cause of death entries for Section 32, Parts I & II, of the standard death certificate are given in Figure 2.

**Figure 3:**
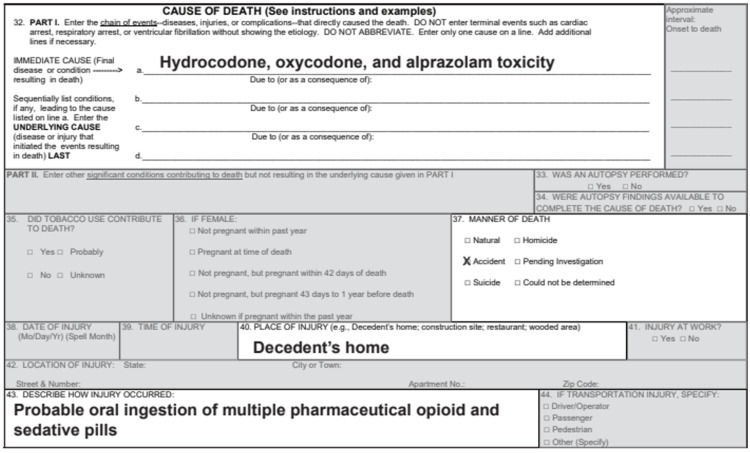
Example of a death certificate Source: CDC [13]; open-source

While Section 32 of the death certificate provides the immediate and contributory causes of death, other sections, notably Sections 32-37, contain useful and relevant information. The CDC’s guidance notes that in a drug-involved death, a toxicological analysis may detect multiple substances but only those in the opinion of the certifying official that is determined to have caused or contributed to the death should be listed [13]. In the example shown in Figure 3, acetaminophen and nicotine were detected along with several other drugs in the toxicology screen of a drug overdose victim but since acetaminophen and nicotine did not cause nor contribute to death, they were not listed as an immediate cause of death [13].

How ICD coding may obscure the true cause of death

Using the example in Figure 2, we see that the CDC’s NVSS codes each drug identified as a cause of death separately according to its ICD T-code (e.g., T40.2 for hydrocodone, T40.2 for oxycodone, and T42.4 for alprazolam). Lost in many fatal drug overdose cases are the true cause of death, namely the additive toxic and often fatal consequences of co-ingesting opioids, benzodiazepines (e.g., alprazolam), and other central nervous system depressants. Studies have linked these drugs, especially when co-ingested in non-therapeutic doses, to increased risk of respiratory depression, coma, and death [18,19].

In a research letter published by the Journal of the American Medical Association (JAMA) in 2018, several government scientists reported that among the 42,249 opioid-related overdose deaths in 2016, 19,413 deaths (45.9%) involved synthetic opioids. Of these deaths, 79.7% involved another drug or alcohol. The most common co-involved substances were another opioid (47.9%), heroin (29.8%), cocaine (21.6%), prescription opioids (20.9%), benzodiazepines (17.0%), alcohol (11.1%), psychostimulants (5.4%), and antidepressants (5.2%) [20].

In drug overdose death cases, significant epidemiologic information is lost when each drug mentioned in the death certificate finds its way into a specific ICD T-code category. Presumably, this enables CDC officials to be able to state a percentage of drug overdose deaths each year in which, for example, T40.2 or T42.4 drugs were involved. However, aggregating data like this obscures not only the prevalence of specific drugs in causing overdose deaths but also ignores entirely the real cause of most fatal overdoses, namely, the additive or toxic interactive effects of polysubstance abuse.

In 2013, CDC scientists published a research letter in which they characterized specific classes of drugs involved in drug-related overdose deaths [21]. More than 20 drug classes were identified by T-codes in a chart showing the number and percentages of times they were mentioned in "pharmaceutical overdose deaths" in 2010 [21]. "Opioid analgesics (T40.2-T40.4)" were involved in 16,651 overdose deaths and "Benzodiazepines (T42.4)" were involved in 5,017 overdose deaths (77.2% of all opioid-involved deaths) [21]. A footnote seemed to acknowledge the limited usefulness of this information: "Deaths are not mutually exclusive. Deaths involving more than one drug or drug class are counted multiple times." [21]. Thus, the death of the hypothetical person whose cause of death information appears in Figure 2, according to the CDC’s methodology for coding drug overdose deaths, would be counted three times - once for each ICD T-coded drug in the death certificate! This limitation, coupled with the CDC’s estimate that 20-30% of death certificates in drug-related cases arrive listing incomplete or imprecise causes, such as "multiple drug intoxication," or "suspected drug overdose," etc., draws our attention to Hanzlick’s concerns.

Becoming a mortality statistic

The path between being certified a death of any cause or manner and becoming a national statistic is a relatively short but important one that typically involves a funeral director, medical certifier (attending physician, medical examiner/coroner, etc.), state vital statistics registrar, and, ultimately, the CDC [13]. As previously mentioned, anonymized death certificate information is shared by state registrars with the CDC [13]. At a minimum, this includes sharing demographic characteristics of the decedent, the time, place, and manner of death, and the literal text of the cause of death as provided by the certifier [13]. Timelines and procedures for reporting deaths and conducting postmortem examinations are set by state law and vary widely among states and sometimes even within states [22].

If a death certificate is not completed properly, the public registrar of vital statistics in the jurisdiction where it is filed may be expected to return it to the certifying official [23]. Multiple studies have shown, however, that death certificate errors are common and certificates rarely are returned to the certifier for correction or completion [24-26]. Death certificate errors have been discussed in the literature since the 1950s, with analyses of data going back to the 1800s [25-28]. Errors and incompletions in death certificates have not been limited to drug overdose cases. For example, studies have shown that ischemic heart disease is vastly overrepresented as a cause of death [29,30]. In a 1998 study of death certificates, it was estimated that the error rate for overestimating coronary heart disease as a cause of death was between 7.9-24.3%, and in older persons, it is as much as twofold [29,31]. Conversely, diabetes and dementia are often under-reported as causes of death [29,30]. In drug overdose cases, when a prescription opioid is believed to be involved, the cause of death frequently is listed as "opioid overdose," regardless of any other aspect of the clinical situation [32].

As noted earlier, 20-30% of drug overdose death certificates arrive at the CDC with the erroneous or incomplete cause of death certifications [33]. Dr. Robert Anderson, chief of the Mortality Statistics Branch of the NVSS, has been quoted as saying that before the current coronavirus pandemic, one in every three death certificates was 'wrong' and that things were about to get worse [14]. This percentage of error is in keeping with the findings of previous studies of the accuracy of death certificate source data [33,34].

Swedish researchers, conducting a metadata assessment of 44 death certificate studies found that methodological variances among certifiers were responsible for different results, some of which were inconsistent with WHO standards [35]. Comparing death certificates with hospital discharge records, they noted a greater risk of certification errors for some diagnostic groups [35]. Malignant neoplasm cases had the highest degree of accuracy, whereas benign and unspecified tumor and chronic obstructive lung disease had the lowest degree of accuracy [36].

Physicians and death certificate errors

Wexelman conducted an anonymous online survey of 531 physician residents in New York City responding to a questionnaire about their experiences in completing death certificates [37]. Only 33.3% believed that cause of death reporting is accurate [37]. Of the 531 respondents, 48.6% admitted to knowingly certifying an inaccurate cause of death [37]. Of respondents who indicated they reported an inaccurate cause of death, 76.8% said that they did so because the system would not accept the correct cause. Of these, 40.5% said admitting office personnel instructed them to "put something else" (as a cause of death), and 30.7% said a medical examiner instructed them to change their initial entry [37]. If the initial cause was rejected by the EDRS software, almost two-thirds (64.6%) of the respondents said that they would instead cite cardiovascular disease, reasoning that everyone dies of cardiac arrest [37].

Education appears to exert a positive effect on improving the accuracy of death certificate information. In a literature review of educational programs designed to improve death certificate accuracy, Aung et al. (2010) concluded that "Pragmatic education on best practice for cause-of-death certification is a basic step to ensure accurate information for each individual case." [38].

While physicians are often the focus of death certificate errors and inaccurate cause of death entries, it is worth noting that they are not the only professionals responsible for completing the death certificate. Funeral directors are responsible for completing the second and third sections of the standard death certificate, as well as making sure the cause of death section has been completed by the appropriate medical or certifying official [39]. Funeral directors are not authorized to complete the cause of death entries reserved exclusively for medical personnel [39]. However, in some states, non-physicians, such as nurse-practitioners and coroners, are authorized to certify a decedent’s cause of death [40]. In Texas, state law requires that a Justice of the Peace must conduct an inquest into the death of someone who dies of suspicious or unusual circumstances, not necessarily requiring autopsies or even viewing the body [41]. The inquest may require a formal autopsy by a medical examiner or forensic pathologist, which are only in larger counties, and a hearing to determine the cause of death [41]. At the conclusion of the inquest, the Justice of the Peace completes the death certificate, including certifying the cause of death [41].

Coroners and medical examiners

Although they share similar medico-legal responsibilities, there are significant differences between medical examiners and coroners. The origin of the coroner system is obscure, but the first mention of it dates to the 12th century [42]. The system of coroner followed England’s colonization around the world [42]. In the US, coroners are elected officials in many jurisdictions and only four states require that they be physicians [43]. According to the CDC, 16 states (and Washington, DC) have centralized medical examiner systems, six states have a county or district-based medical examiner system, 14 states have a county-based system with a mixture of coroner and medical examiner office, 14 states have a county-, district-, or parish-based coroner system, and 25 states solely have state medical examiners [44]. Qualifications to become a coroner differ among states. In Georgia, to run for the office of coroner one must be at least 25 years of age, a registered voter, a high school graduate, and not have a felony conviction [45]. In 2016, of Georgia’s 154 coroners, only one was a physician and four had criminal records [45]. According to the Atlanta Journal-Constitution (AJC), the state’s largest newspaper, the job of coroner is considered a part-time job in Georgia and the AJC’s review of state records showed coroners listing their other occupations as farmer, car wash owner, hairdresser, plumber, etc. [45]. The AJC examined thousands of death certificates issued statewide since 2011 and found almost three dozen cases in which coroners ruled that shooting victims died of “natural causes.” [45]. In nearly four of every 10 cases ruled a suicide, no autopsy was performed to confirm the cause and manner of death [45]. Although coroners who are not physicians cannot perform autopsies, they are responsible for making the initial decision for when an autopsy is required. If the initial decision is erroneous, as mentioned in the examples of suicide reported by the AJC investigation, the result is a loss of the data for which the death certification system was created in the first place.

The US is not alone in tolerating these conditions. Kelsall and Bowes [46] reported that in Canada, "(T)here is no accreditation system for coroner or medical examiner offices, no national standards for the investigation or classification of death, no nationally recognized training program or credentialing system for coroners and medical examiners, and no agreement on common outcome measures against which to evaluate performance." Autopsy requirements vary widely among Canadian provinces and the authors, both physicians, contend that "assigning deaths as 'undetermined' in cases of drug overdose, for example, because an autopsy was not done, precludes efforts to prevent future deaths." [46].

International Classification of Diseases

Technically known as the International Statistical Classification of Diseases and Related Health Problems, the ICD is considered by the WHO to be the world’s "bedrock for health statistics." [47]. As the WHO describes the ICD, "It maps the human condition from birth to death: any injury or disease we encounter in life − and anything we might die of − is coded." [48]. While coding, that is, the assigning of alphanumeric codes to diseases and injuries represents the core genius of the ICD for harmonizing global health statistics, it also may hamper the proper classification of important information when coding categories and guidelines are too restrictive. Originally developed to classify mortality and to provide a coding schema for reporting causes of death, the ICD has expanded over the years to include classifying morbidity and many other items, services, and procedures related to the delivery of healthcare [48].

**Figure 4:**
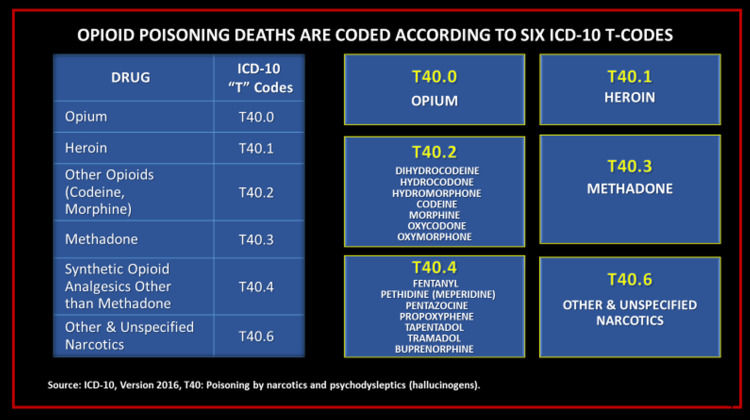
ICD codes for opioid poisoning Source: WHO [50]; open-source

In the US, the ICD consists of two components, identified as ICD-10-CM, for clinical modifications, and ICD-10-PCS, for procedural coding systems [48]. The NCHS, with guidance from the Centers for Medicare and Medicaid Services, has responsibility for developing the ICD-10’s clinical modifications used in the US [48]. In 1999, the final year for using ICD-9, there were 13,000 diagnostic codes in the US clinical modifications version [49]. ICD-10 that followed ICD-9 included 68,000 codes in its clinically modified version - a fivefold increase [50]. Chapter 19 of the ICD-10 established two subcategories identified as S and T codes [51]. The S codes are for various single body region injuries, and the T codes cover injuries to unspecified body locations, poisonings, and other external consequences [51]. Containing more than 40,000 individual codes for characterizing health-related topics and items, the ICD has just six T-codes for identifying all opioids: T40.0 (opium), T40.1 (heroin), T40.2 (other natural and semisynthetic opioids, including morphine, codeine, oxycodone, hydrocodone, hydromorphone, and oxymorphone), T40.3 (methadone), T40.4 (synthetic opioids other than methadone, including fentanyl, meperidine, pentazocine, propoxyphene, tapentadol, buprenorphine, and tramadol), and, finally, T40.6 (other and unspecified narcotics) (Figure 4) [50].

CDC finally comes clean - maybe!

In 2018, four senior CDC analysts, including the head of the Epidemiology and Surveillance Branch, published a three-page editorial acknowledging that the number of US deaths in 2016 attributed to prescription opioid overdoses was erroneously overstated [51]. The problem was caused by including IMF in the coding category - T40.4, synthetic opioids - for the prescribed version: "Thus, rates of prescription opioid-involved deaths estimated with the traditional method may have been inflated in recent years because of the increase in death rates involving synthetic opioids (e.g., fentanyl)." [52]. According to internal CDC reports, the IMF problem was discovered during the analysis of 2015 data being prepared for the 2016 report of prescription opioid overdose deaths [*Courtney Lenard, Public Affairs Officer, CDC. Email to Puja Seth, Chief, Epidemiology and Surveillance Branch, CDC, dated March 27, 2018, Subject: Interview Request (talking points) *(unpublished); Obtained by John J. Coleman on January 20, 2021,via Freedom of Information Act Request re: Case 21-00194-FOIA,along with *the letter from HHS signed Roger Andoh, FOIA Officer *(unpublished*; *available from the correspondence author of this article upon reasonable request). 2018.]. Although it took two years for the problem to be explained in a journal article, the CDC was aware of IMF long before a sudden spike in deaths attributed to synthetic opioids in 2015 was traced to IMF [53]. In 2008, the CDC published a report of what it called "Nonpharmaceutical Fentanyl-Related Deaths," a form of illicitly produced fentanyl that appeared in multiple US states during the period from April 2005 to March 2007 [54]. Nonpharmaceutical fentanyl, according to the 2008 CDC report, was responsible for more than a thousand deaths in the US over the course of two years [54]. In 2013, IMF reappeared causing deaths in the northeast [55]. Rhode Island authorities notified the CDC that acetyl fentanyl, a fentanyl analog up to five times as potent as fentanyl, had been identified in 10 drug overdose deaths in the state between March 7 and April 11, 2013 [56]. During and shortly after the month-long investigation in Rhode Island, four more overdose deaths occurred [56]. Besides the Rhode Island deaths, a CDC field report at the time cited a cluster of 50 similar IMF deaths reported by authorities in Pennsylvania [56]. In their editorial, the CDC analysts reported that until 2016, the NVSS calculated annual prescription opioid overdose deaths by summing deaths coded T40.2, T40.3, and T40.4 [57]. The latter code - T40.4 - was identified as the source of error in the 2016 data [58]. All mentions of fentanyl - including non-prescribed IMF - were coded T40.4 by the agency’s nosologists [57]. In 2016, the sum of the three codes amounted to 32,445 deaths [58]. This figure, the CDC analysts acknowledged, was erroneous because it included deaths involving IMF that were mistakenly coded and counted as a "prescribed" synthetic opioid [57,58]. To correct the error, the CDC analysts proposed what they called a 'conservative' method for re-calculating prescription opioid overdose deaths in 2016 and prior years [57]. Their conservative method simply removed all deaths coded T40.4 from the count [57]. This, in turn, reduced the number of prescription opioid overdose deaths in 2016 from 32,445 to 17,087 - a sizable drop of 47.3% [57]. The analysts candidly conceded, however, that the "conservative" approach likely produced an undercount error because by deleting all T40.4 deaths, they were removing an unknown number of deaths caused by, or involving, prescription fentanyl, as well as deaths caused by, or involving, other prescription opioids identified with the same T40.4 code (e.g., meperidine, pentazocine, propoxyphene, tapentadol, buprenorphine, and tramadol) [57].

**Figure 5:**
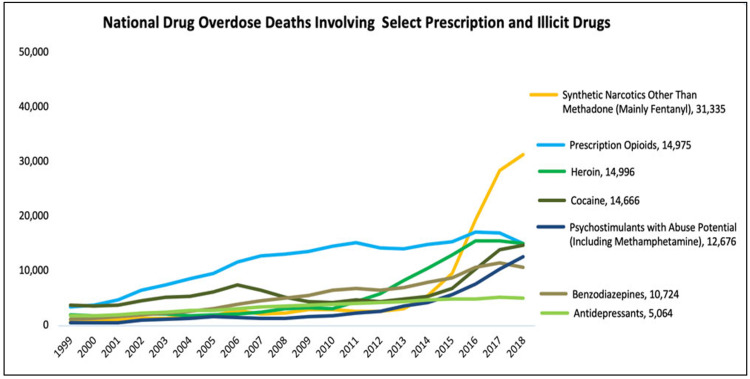
Data of the National Institute of Drug Abuse Source: NIDA [59]; open-source

The discovery of the T-code error in the CDC’s prescription opioid overdose death figures for 2016 prompted us to search for other examples of similar errors in the CDC’s coding system for controlled substances. We discovered that benzodiazepines, a class of drugs often associated with fatal opioid drug overdoses, are undifferentiated in ICD T-codes [59]. More than a dozen FDA-approved benzodiazepine drugs are identified by a single ICD T-code (T42.4) [59]. In 2018, according to the National Institute on Drug Abuse, benzodiazepines were involved in 15.8% of all drug overdose deaths (Figure 5) [60]. While the aggregate coding of all benzodiazepines is not an error, per se, it does reduce the epidemiological value and specificity of the data. In their 2018 article, the CDC analysts did not acknowledge these limitations or other miscoding errors for drugs other than IMF. We, however, discovered similar anomalies in the CDC’s use of the ICD for reporting non-opioid overdose mortality. For example, cocaine, a controlled substance that is FDA-approved for medical use, is also manufactured illicitly and sold on the street as cocaine or cocaine base (also known as "crack") [61,62]. Despite important epidemiological differences between the licit and illicit forms of cocaine, all references to this drug in the ICD are categorized under a single T-code (T40.5) [59].

Methadone: prescribed for pain but administered/dispensed for opioid use disorder (OUD)

While investigating the topic, we discovered a more serious source of error involving the CDC’s handling of methadone-related overdose deaths. Methadone is a Schedule II opioid agonist with dual indications for the management of severe chronic pain and for treating opioid use disorder (OUD) [63]. When used for pain treatment, methadone is subject to the same regulations as any other Schedule II prescription drug. However, when used for treating OUD, federal law prohibits the prescribing of methadone [64,65]. Methadone used for treating OUD must be administered or dispensed by an authorized practitioner in an opioid treatment program (OTP) certified by the Substance Abuse and Mental Health Services Administration (SAMHSA) and registered by the Drug Enforcement Administration (DEA) [63,66,67]. Patients admitted for OUD treatment with methadone receive the medication under the direct supervision of a practitioner authorized to dispense or administer (but not prescribe) the medication to the patient in liquid oral form. Patients must visit the OTP for the first 90-days to be administered daily doses of methadone. After this period, take-home doses of methadone may be approved for dispensing to the patient by the OTP, provided that certain criteria are met [66,67]. During the COVID-19 pandemic, SAMHSA relaxed take-home rules to allow states with declared health emergencies to request an exemption for stable methadone patients, regardless of time in the program, to receive as much as 28 days of take-home doses of methadone [68]. While necessary, take-home methadone has been identified with diversion and misuse because of the drug’s high street value [69]. According to the director of the largest opiate treatment program in Baltimore, a 28-day supply of diverted methadone is worth as much as $2,000 on the street [69].

**Figure 6:**
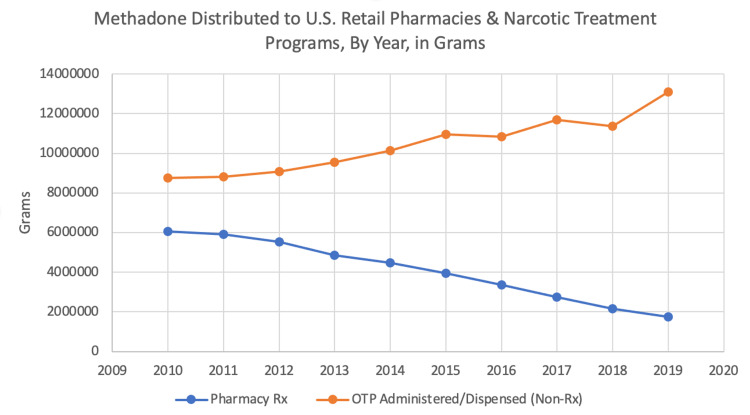
Methadone official consumption in the US Data for graph sourced from: DEA [71]

 In 2014, according to the CDC, methadone accounted for approximately 1% of all opioids prescribed for pain but was involved in approximately 23% of all prescription opioid deaths [70]. The CDC did not report how many methadone deaths resulted from the prescribed form (*i.e.*, for pain), and how many methadone deaths resulted from the nonprescribed form (i.e., administered/dispensed for OUD treatment). Citing no evidence other than the total number of deaths from methadone, a CDC report in 2017 claimed that "the preponderance of methadone-associated morbidity and mortality likely arises from its use for pain." [70]. This assumption, however, is not supported by the record. Prescribed methadone, dispensed by retail pharmacies, in the US decreased 71.2% between 2010 and 2019 (from 6,068,686.51 grams to 1,746,684.03 grams) [71]. During the same period, the volume of nonprescribed methadone administered/dispensed by OTPs increased 49.9% (from 8,746,056.41 grams to 13,114,262.44 grams) [71]. In 2017, the year that the CDC reported the data "the preponderance of methadone-associated morbidity and mortality likely arises from its use for pain," the volume of nonprescribed methadone administered or dispensed by OTPs was more than fourfold the volume of methadone prescribed for pain (11,686,565 grams administered/dispensed by OTPs vs. 2,740,641 grams prescribed for pain) (Figure 6) [71].

The data in Figure 6 show what appears to be an inverse relationship in the use of methadone between the two populations, i.e., chronic pain patients and OUD patients. Given what is known about ICD coding and how its use skewed CDC mortality figures for prescription opioid overdose deaths in 2016, the subject of how the CDC codes methadone-involved deaths demand scrutiny. It seems like CDC’s annual tally of prescription opioid overdose deaths, which includes all methadone-involved deaths, continues to be skewed by including deaths involving non-prescribed methadone.

The CDC ignored error signals in calculating prescription opioid overdose deaths

In their 2018 article, the CDC analysts (Seth et al.) provided a table showing the calculation of prescription opioid overdose deaths from 1999 to 2016, describing what they termed a "conservative" approach and a "traditional" approach (Table 1) [52]. By 2013, the number of deaths in the T40.4 category (see the column in Table 1 titled, "Synthetic opioids, other than methadone") began to increase sharply after several years of relative stability [52]. By 2016, this category had increased over 525% (from 3,105 deaths in 2013 to 19,413 deaths in 2016) [52]. By 2016, the CDC’s data were showing that more than 37.4% of all prescription opioid deaths were being caused by "synthetic opioids other than methadone," the T40.4 category that besides fentanyl, includes pethidine (meperidine), pentazocine, propoxyphene, tapentadol, tramadol, and buprenorphine [52]. The CDC findings regarding T40.4 drugs were inconsistent with prescribing data available at the time showing that the volume of fentanyl dispensed by retail pharmacies in the US declined between 2013 and 2016 by 13.2% (from 403,773.3 grams to 350,397.3 grams) [72]. In the past, CDC analysts have described a close linear relationship between sales volumes of opioids and opioid overdose deaths, showing that whenever sales increase, they are followed by proportional increases in overdose deaths [73-76]. If one assumes that the converse of this also is true, i.e. a decrease in opioid sales is associated with fewer overdose deaths, there should have been a decrease - not an increase - in prescription fentanyl overdose deaths for the period in question.

**Table 1:** Prescription opioids overdose deaths in US (2000-2016), including and excluding synthetic opioids Deaths are classified according to the ICD-10. Approximately 1/5 of drug poisoning deaths lack information on the specific drugs involved. Data sourced from (with permission): Seth et al. [52]

	Conservative definition for prescription opioids: natural and semisynthetic opioids and methadone		Traditional definition for prescription opioids: natural and semisynthetic opioids and methadone, and other synthetic opioids		Synthetic opioids other than methadone	
Year	Number	Overdose deaths per 100,000	Number	Overdose deaths per 100,000	Number	Overdose deaths per 100,000
2000	3785	1.3	4400	1.5	782	0.3
2001	4770	1.7	5528	1.9	957	0.3
2002	6483	2.3	7456	2.6	1295	0.4
2003	7461	2.6	8517	2.9	1400	0.5
2004	8577	2.9	9857	3.4	1664	0.6
2005	9612	3.2	10928	3.7	1742	0.6
2006	11589	3,9	13723	4.6	2707	0.9
2007	12796	4.2	14408	4.8	2213	0.7
2008	13149	4.3	14800	4.8	2306	0.8
2009	13523	4.4	15597	5.0	2946	1.0
2010	14583	4.7	16651	5.4	3007	1.0
2011	15140	4.9	16917	5.4	2666	0.8
2012	14240	4.5	16007	5.1	2628	0.8
2013	14145	4.4	16235	5.1	3105	1.0
2014	14838	4.6	18893	5.9	5544	1.8
2015	15281	4.7	22598	7.0	9580	3.1
2016	17087	5.2	32445	10.2	19413	6.2

By 2013, the number of deaths in the T40.4 category (see the column in Table 1 titled, "Synthetic opioids, other than methadone") began to increase sharply after several years of relative stability [52]. By 2016, this category had increased over 525% (from 3,105 deaths in 2013 to 19,413 deaths in 2016) [52]. By 2016, the CDC’s data were showing that more than 37.4% of all prescription opioid deaths were being caused by "synthetic opioids other than methadone," the T40.4 category that besides fentanyl, includes pethidine (meperidine), pentazocine, propoxyphene, tapentadol, tramadol, and buprenorphine [52]. The CDC findings regarding T40.4 drugs were inconsistent with prescribing data available at the time showing that the volume of fentanyl dispensed by retail pharmacies in the US declined between 2013 and 2016 by 13.2% (from 403,773.3 grams to 350,397.3 grams) [72]. In the past, CDC analysts have described a close linear relationship between sales volumes of opioids and opioid overdose deaths, showing that whenever sales increase, they are followed by proportional increases in overdose deaths [73-76]. If one assumes that the converse of this also is true, i.e*.* a decrease in opioid sales is associated with fewer overdose deaths, there should have been a decrease - not an increase - in prescription fentanyl overdose deaths for the period in question.

**Figure 7:**
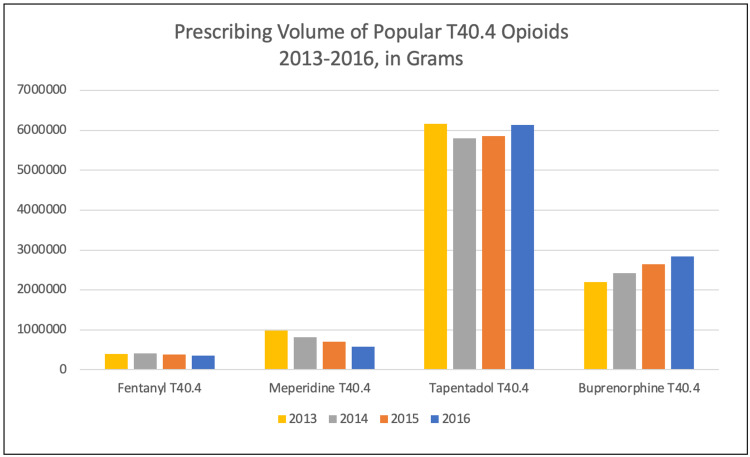
Official data on sales of some opioids in the US Source: DEA ARCOS [71]; open-source

 Instead, according to Table 1, three of every five drug overdose deaths (19,413/32,445 = 59.8%) in 2016 were coded T40.4 ("Synthetic opioids, other than methadone"). A rapid increase in overdose deaths caused by T40.4 drugs, a category that includes fentanyl and several other scheduled opioids, simply made no sense at a time when the prescribing of all T40.4 drugs, including fentanyl, was stable or declining (Figure 7).

Buprenorphine, the only T40.4 drug showing an increase during this time, like methadone, has a dual indication for treating pain and OUD [77]. Because it is a Schedule III drug, physicians can apply for authorization to prescribe buprenorphine for the treatment of OUD [78,79]. DEA distribution records do not differentiate between buprenorphine prescribed for treating pain and buprenorphine prescribed for treating OUD. Given the expansion of out-patient treatment of OUD with buprenorphine in the last decade, it is reasonable to assume the increased volume of prescribed buprenorphine depicted reflects the increased prescribing of the drug for OUD treatment [80].

Discussion

The modern death certificate may have taken five centuries to create, but, as our study is showing, some of the problems that plagued its earliest versions continue today. The decentralization of authority for certifying and reporting deaths has produced substantial differences in how causes of death are characterized and reported. By any measure, the error rate in death certifications, especially in drug-involved cases, remains excessive. It is critical that death data are accurate for obvious reasons. If data used by the CDC have significant error rates, which appears to be the case, there needs to be adjustments made to improve the accuracy of this program. We are not the first authors to suggest this; errors in mortality causes and reporting, as noted above, have been described in the literature for decades. 

The US government reports that health expenditures in 2017 amounted to $3.4 trillion, a sizable portion of which was spent on disease prevention and control [81]. When the source data for achieving public health goals are less than accurate, as in the situations highlighted in this paper, policymakers and public health officials are likely to focus attention and resources on the wrong things - such as the sources for prescribed fentanyl instead of the sources for illicitly manufactured non-prescribed fentanyl and fentanyl analogs. Mortality resulting from cardiovascular disease may be imprecisely overstated because of software limitations and input errors. As briefly mentioned earlier, reports of COVID-19 deaths according to WHO guidelines appear inconsistent and unreliable among reporting nations. All these problems begin but do not end with the death certificate.

In 2018, the same year that the CDC finally admitted that its system for tabulating prescription opioid overdose deaths was flawed, Congress enacted the Support for Patients and Communities Act [82]. The 250-page Act contained more than a hundred references to drug overdose prevention [82]. In Subtitle Q, Sect. 392A, "Preventing Overdoses of Controlled Substances," the CDC was directed to coordinate "controlled substance overdose data collection" by, among other things, "(C) Modernizing the system for coding causes of death related to controlled substance overdoses to use an electronic-based system." [82]. This provision likely was intended to have the CDC correct the present flawed system for coding causes of death related to drug overdoses before the next update of the ICD, an all-electronic online version, expected to debut in January 2022 [83].

The Act also mandated the reinstatement of the Drug Abuse Warning Network (DAWN), a retrospective survey of drug-related hospital admissions that inexplicably was halted by SAMHSA at the height of the opioid abuse crisis in 2011 [82]. Well into two years since these important improvements were statutorily ordered by Congress, neither is yet functional (although a contract to reinstitute the DAWN program was awarded in November 2018 to Westat Inc. (Rockville, MA), its former vendor [84]). The Department of Health and Human Services, the parent agency for the CDC and SAMHSA, has opted instead to provide grants to state agencies to perform tasks assigned by federal law to the CDC, namely, to compile drug-involved mortality statistics with a greater emphasis on expanding the specificity and characteristics of drug-involved overdose deaths. Thus far, the CDC has recruited and funded about 47 states to provide this information that the CDC gathers, reformats, and publishes under its own rubric [72].

Why the CDC ignored for years clear signals that its methodology for calculating prescription opioid overdose deaths was flawed is unknown. It seems like, even today, the CDC has no way of determining the actual number of prescription opioid overdose deaths each year. For more than a decade, the CDC’s erroneous reports of prescription opioid overdose deaths went unchallenged while being used by Congress and the Executive Branch as the justification for public policy. In just nine years, from 2012 to 2020, the federal government expended $261.3 billion for drug control [85]. It is estimated that the states spent at least as much, if not more, for the same purpose. The full effect of the CDC’s reporting failures as discussed herein may never be known. What is known is that the amount of money spent on drug control in the US has continued to increase along with the magnitude of the problem. An improved system that is responsive and accurate would likely produce better results. Moreover, it would generate less confounding scientific literature, which most of the time is based on US official data.

It should be noted that in 2021 (beginning October 1, 2020), CMS finalized clinical modifications to specifically identify poisonings by fentanyl or fentanyl analogs within the T40.4 coding category [86]. The same treatment was applied to poisonings by tramadol [86]. Unfortunately, these changes did nothing to remedy the IMF problem because they did not offer a specific coding definition for distinguishing illicit from licit fentanyl. Actually, they were responsible for misinformation appearing in the White House report [87]. Definitely, it is a difficult problem to fix.

Limitations

This narrative review has several limitations. Amongst others, the US situation, which was described in detail, was not compared with reports from the rest of the world. As previously stated, the huge majority of the scientific literature on this topic derives from US official data. The 117 nations that use the ICD for reporting mortality may experience similar limitations in the coding of drug-involved deaths. The CDC’s problem directly results from its use of the ICD codes to categorize drug-involved mortality. Presumably, any other country presently using the same ICD system and encountering counterfeit opioids or benzodiazepines (mostly alprazolam), or using methadone for opiate addiction treatment by administration/dispensing-only (not by prescription except for pain treatment) - would obviously encounter the same problems as the CDC unless, that is, they used more sensitive reporting elements to properly identify drug deaths by specific drugs rather than general categories. The CDC has begun to do this but only from about 2016 when it discovered the error caused by including IMF in its compilation of prescription opioid overdose deaths. Considering that in other countries too, the official data are prevalently based on ICD, it would be important (and interesting) to compare most of the aspects of the cross-pollination, and its influence on the local political and social choices.

## Conclusions

The CDC’s problems in reporting drug-involved mortality begin with the source data. Drug-involved overdose deaths pose a unique problem for medical examiners and coroners because of the unavoidable delay in receiving postmortem toxicology results. We found that this problem is not unique to drug overdose deaths, and problems involving deaths caused by other causes/diseases affecting the accuracy of death certificates are also common. The recent pandemic has raised additional questions as to how nations interpret WHO guidelines in recording COVID-19 mortality. 

Why it took more than a decade and several problems before the CDC was finally called to task to answer questions about the issues addressed in this paper is not yet entirely clear. The CDC’s solution for addressing the IMF problem was not a solution at all but simply a way to produce a smaller inaccurate number. Prescription drug abuse remains a national crisis and prescription opioids taken for non-therapeutic purposes contribute to tens of thousands of overdose deaths every year. Reducing drug-involved mortality will likely elude us until we have trustworthy systems that provide reliable data unobscured by antiquated algorithms and codes better suited to conditions of a bygone era.
